# Drug delivery dynamics dictate evolution of bacterial antibiotic responses

**DOI:** 10.1093/ismejo/wraf082

**Published:** 2025-05-11

**Authors:** John C Crow, Hao Geng, Christopher J Geiger, Timothy J Sullivan, Shannon M Soucy, Daniel Schultz

**Affiliations:** Department of Microbiology & Immunology, Dartmouth – Geisel School of Medicine, Hanover, NH 03755, United States; Department of Microbiology, Immunology & Molecular Genetics, University of California Los Angeles, Los Angeles, CA 90095, United States; Department of Microbiology & Immunology, Dartmouth – Geisel School of Medicine, Hanover, NH 03755, United States; Department of Microbiology & Immunology, Dartmouth – Geisel School of Medicine, Hanover, NH 03755, United States; Department of Biosciences, Rice University, Houston, TX 77005, United States; Department of Biomedical Data Science, Dartmouth – Geisel School of Medicine, Hanover, NH 03755, United States; Department of Biomedical Data Science, Dartmouth – Geisel School of Medicine, Hanover, NH 03755, United States; Department of Microbiology & Immunology, Dartmouth – Geisel School of Medicine, Hanover, NH 03755, United States

**Keywords:** microbial evolution, antibiotic resistance, gene regulation

## Abstract

Microbes inhabit natural environments that are remarkably dynamic. Therefore, microbes harbor regulated genetic mechanisms to sense shifts in conditions and induce the appropriate responses. Recent studies suggest that the initial evolution of microbes occupying new niches favors mutations in regulatory pathways. However, it is not clear how this evolution is affected by how quickly conditions change (i.e. dynamics), or which mechanisms are commonly used to implement new regulation. Here, we perform experimental evolution on continuous cultures of *Escherichia coli* carrying the tetracycline resistance *tet* operon to identify specific mutations that adapt drug responses to different dynamic regimens of drug administration. We find that cultures evolved under gradually increasing tetracycline concentrations show no mutations in the *tet* operon, but instead a predominance of fine-tuning mutations increasing the affinity of an alternative efflux pump AcrB to tetracycline. When cultures are instead periodically exposed to large drug doses, all populations evolved transposon insertions in repressor TetR, resulting in loss of regulation and constitutive expression of efflux pump TetA. We use a mathematical model of the dynamics of antibiotic responses to show that sudden exposure to large drug concentrations overwhelm regulated responses, which cannot induce resistance fast enough, resulting in selection for constitutive expression of resistance. These results help explain the frequent loss of regulation of antibiotic resistance by pathogens evolved in clinical environments. Our experiment supports the notion that initial evolution in new ecological niches proceeds largely through regulatory mutations and suggests that transposon insertions are the main mechanism driving this process.

## Introduction

To enable the colonization of ecological niches where conditions change frequently [1, 2], microbes are equipped with inducible mechanisms that sense changes in their surroundings and initiate the appropriate cellular programs [3]. In some cases, responses are not time-sensitive and mostly tune gene expression to optimal levels, such as when cells respond to shifts in nutrient conditions [4, 5]. However, when cells are exposed to antibiotics or other harmful compounds, cell survival depends on the quick deployment of its defenses, while gene expression is still possible [6, 7]. Therefore, even when microbes carry mechanisms to deal with hostile environments, sudden and frequent changes in conditions still threaten cells that are too slow to respond.

Dynamic environments, where conditions shift rapidly, pose fundamentally different selective pressures on the evolution of antibiotic responses [8–10]. Antibiotic resistance mechanisms originate in the soil, where antibiotic concentrations are typically low [11], within a selection window that inhibits the growth of sensitive strains while enriching resistant subpopulations carrying beneficial mutations [12, 13]. In contrast, antibiotics are used in the clinic in high doses, with the intent of wiping out entire microbial populations [14, 15]. Such extreme environments pose strict bottlenecks, resulting in selective sweeps of any surviving mutants [16, 17], however unfit [18, 19]. However, although much attention has been devoted to the evolution of antibiotic resistance under steady drug concentrations [20–22], we still lack an understanding of the unique challenges posed by variable environments. Most studies in variable environments focus on how evolution proceeds under shifting demands and are typically implemented by occasional changes in conditions that are otherwise steady [23, 24]. Here, we focus instead on the dynamics of the environment itself as an evolutionary pressure, where drug concentrations are high and change quickly. Recent studies suggest that the short-term evolution of microbes in such challenging environments relies heavily on mutations affecting regulatory pathways [25–27].

**Figure 1:**
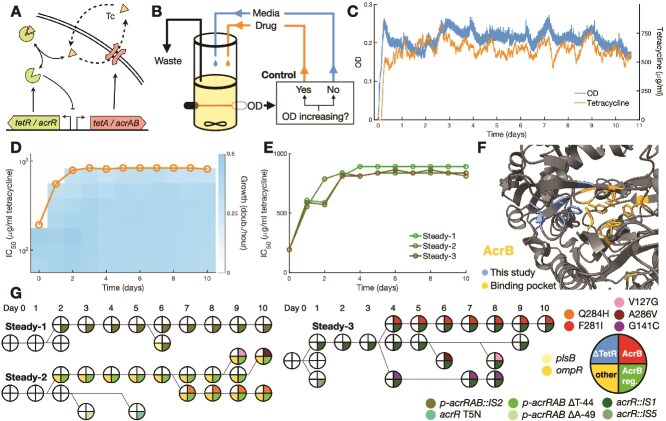
Steady drug regimen favors mutations in the AcrB binding pocket. (A) *Tet* and *acr* resistance mechanisms: Tetracycline (Tc), a translation inhibitor, diffuses across the cell membrane and binds transcription repressors TetR and AcrR, which become inactive and release expression of efflux pumps TetA and AcrAB, which export tetracycline out of the cell. Although *tet* resistance is specific for tetracycline, *acr* is not. (B) Continuous culture setup with media and drug control for the steady environment. If OD increased over the previous half hour, a fixed drug dose is added to the culture. (C) Progression of OD and drug concentration for population Steady-2 over the course of the experiment. (D) Growth rates measured in samples of population Steady-2 from each day of the experiment along a gradient of tetracycline. Circles indicate the drug concentrations at which growth is reduced to half (IC_50_ concentration). (E) Increase in resistance for each steady population, measured by the IC_50_ concentration. (F) Locations of AcrB mutations detected in evolved populations within the protein structure. We found several mutations around the drug-binding site. (G) Cladograms of isolates from each steady population, showing the presence of high-abundance mutations. Five isolates were picked from each population in each day, and presence of select mutations was confirmed by sequencing. Phylogenetic relationships were inferred from the co-occurrence of each mutation in each isolate.

To study the evolution of antibiotic responses, we focus on tetracycline resistance in *Escherichia coli*. Although tetracycline is not typically used to treat *E. coli* infections in humans, *E. coli* from the gut microbiota is affected by tetracycline treatments and tetracycline resistance is used as an indicator to monitor antibiotic resistance [28]. Tetracycline resistance is mediated by two inducible efflux mechanisms capable of transporting the drug out of the cell—the *tet* and *acr* operons [29, 30] (Fig. 1A). Although the *acr* operon transports a wide variety of toxic compounds and is part of the *E. coli* core genome [31, 32], the *tet* operon is a tetracycline specific module of the *E. coli* pan-genome that provides the bulk of resistance in strains where it is present [33, 34]. Both mechanisms are regulated by repressors of the same family—TetR and AcrR, respectively [35, 36]—that can bind tetracycline and lose affinity for DNA, releasing expression of their respective efflux pumps, *tetA* and *acrAB*. Because the *acr* operon is not optimized for tetracycline efflux, several mutations in *acrB* have been reported to increase tetracycline resistance [37, 38]. Additionally, active tetracycline transport via TetA involves an ion exchange that has been shown to disrupt the membrane potential, thereby posing a trade-off between resistance and toxicity in *tetA* expression [39]. Ultimately, tetracycline resistance depends on the interaction between these two inducible mechanisms, which differ in costs/benefits and drug specificities. Resistance can be increased by acquiring mutations in either one.

Here, we use a system of automated continuous cultures to experimentally evolve tetracycline resistant *E. coli* populations under different regimens of drug administration. We compare evolution under a steady drug environment, where the drug concentration changes gradually, with a dynamic environment where the population is periodically subjected to sudden exposures to high drug concentrations.

## Materials and methods

### Media, drugs, and strains

All experiments were conducted in M63 minimal medium (2 g/l (NH_4_)2SO_4_, 13.6 g/l KH_2_PO_4_, 0.5 mg/l FeSO_4_, 7H_2_O) supplemented with 0.2% glucose, 0.01% casamino acids, 1 mM MgSO4, and 1.5 mM thiamine. Tetracycline and IPTG solutions were freshly made from powder stocks (Sigma) and filter-sterilized before each experiment. As the ancestral strain, we used *E. coli* K-12 strain MG1655 *rph* + Δ*LacIZYA*, with the native *tet* resistance mechanism from the Tn*10* transposon integrated into the chromosome at site HKO22 with a pIT3-CH integrating plasmid.

### Setup of continuous cultures

Glass vials were coated with Sigmacote (Sigma) to prevent biofilm growth. Vials, vial heads, tubing, and all connections were autoclaved before assembly. Complete sterilization was ensured by running 10% bleach, 70% ethanol, and sterile water consecutively through all tubing. Experiments were carried out at 37°C in sterile M63 media (as described above). Drug media consisted of M63 media with tetracycline added to 1000 μg/ml, mixed until completely dissolved in solution, and filter sterilized into the container used for the experiment. Vials were filled with 15 ml of M63 and used to blank OD measurements.

### Continuous culture experiments

The experiment consisted of two experimental groups (Dynamic and Steady); each group was composed of three replicate vials. Samples were taken daily and stored as glycerol stocks in a 96 well plate at −80°C. Vials were switched daily to prevent biofilm growth.

### Steady regimen

The steady group was run as a morbidostat. We used 15 ml of cell cultures at a fixed dilution rate of 0.35 h^−1^, with media added every 5 min, which corresponds to a growth rate of 0.5 doublings per hour and an OD (optical density) of 0.15 for the ancestral strain in the absence of drug. Tetracycline was automatically added to the cultures in increments of 4.4 μg/ml in every cycle where the population showed net growth over the previous 30 min. This regimen initially resulted in cell densities between 0.2 and 0.3, while keeping a constant selective pressure resulting from drug inhibition of growth. After 3 days, all 3 populations were growing stably with OD around 0.3 and drug concentrations above 500 μg/ml, which is ~5-fold higher than the minimum inhibitory concentration (MIC) of the ancestral WT strain.

### Dynamic regimen

**Figure 2:**
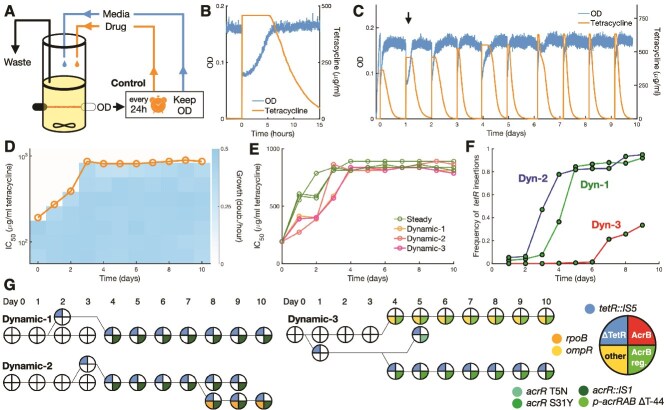
Dynamic drug regimen favors transposon insertions in the *tetR* gene. (A) Continuous culture setup with media and drug control for the dynamic environment. Cultures are suddenly exposed to a large drug dose every 24 h but are otherwise maintained at a target OD by dilution with fresh medium. (B) Cultures are exposed to tetracycline by the addition of a large volume of medium containing the drug, which reduces OD to half. Dilution with fresh medium resumes when the population doubles to again reach the target OD. (C) Progression of OD and drug concentration for population Dynamic-2 over the course of the experiment. The arrow indicates the exposure depicted in (B). (D) Growth rates measured in samples of population Dynamic-2 from each day of the experiment along a gradient of tetracycline. Circles indicate drug concentrations at which growth is reduced to half (IC_50_ concentration). (E) Increase in resistance for each population, measured by the IC_50_ concentration. Acquisition of resistance is slower in dynamic populations. (F) Prevalence of transposon insertions in *tetR* over time in each dynamic population. Such mutations were the first to be observed in all dynamic populations. (G) Cladograms of isolates from each dynamic population showing the presence of high-abundance mutations. Five isolates were picked from each population in each day, and presence of select mutations was confirmed by sequencing. Phylogenetic relationships were inferred from the co-occurrence of each mutation in each isolate.

The dynamic group was run as a modified turbidostat. We kept 15 ml of cell culture at a fixed target OD of 0.15 by the controlled dilution of the culture with drug-free media, delivered every 5 min. At 24 h intervals, cultures were exposed to a single large dose of tetracycline by the addition of 15 ml of medium with drug, which also caused the population to be diluted by half. We then waited for the culture to recover to the target OD without dilution. Following recovery, controlled addition of fresh medium resumed, and the drug concentration gradually decreased to zero (Fig. 2B). We started with exposure to 150 μg/ml of tetracycline on the first day, which was shown to cause a ~ 5 h delay in recovery from drug exposure in the ancestral WT population. The drug concentrations for subsequent exposures were adjusted by a factor of $5/{t}_r$, where ${t}_r$ is the recovery time in hours, which resulted in an increase in drug concentration if the recovery time was shorter than 5 h. If a culture did not fully recover within 24 h, no drug was added to that cycle.

All three populations quickly recovered from the first exposure and were followed by significant increases in drug dose for the exposures in the following days. Recovery times were longer and more variable within the first few days, but eventually stabilized at around 45 to 60 min by the end of the experiment (Fig. 2C, SI). In general, populations recovered quickly and showed fast growth following drug exposures, which resulted in fast dilution rates and ensured that the drug was removed from the culture within 10 h.

### Genomic sequencing of evolved populations

Samples were collected daily during the experiment. On completion, gDNA from each final culture was extracted and sent for whole genome sequencing, together with the ancestral WT population (MG1655 *tet+*), to identify adaptive mutations by aligning WGS reads to a reference genome. We also sequenced a culture evolved in a turbidostat in the absence of drug to identify possible mutations that are adaptive to growth under our experimental conditions, but not related to drug resistance. We did not find any significant such mutations.

Glycerol stocks from the final evolved populations were inoculated 1:100 in 5 ml LB and grown for five hours. Samples were pelleted and DNA was extracted using DNEasy Kits (Qiagen). Whole genome sequencing, producing a median of 2.3 million, 2×151bp, paired-end reads, was performed by the Genomics Shared Resource, and sequencing analysis was carried out by the Genomic Data Science Core at Dartmouth College.

### Analysis of whole genome sequencing

Raw reads were trimmed using Trimmomatic v.0.39 [40] and the following parameters: “ILLUMINACLIP:TruSeq3-PE-2.fa_wpoly.fa:2:30:10:2:keepBothReads LEADING:3 TRAILING:3 MINLEN:36”. Reads were subsequently aligned to the *E. coli* K-12 U00096.2 reference genome using BWA v.0.7.17 [41]. Single nucleotide variants and small insertions or deletions were called using FreeBayes v.1.3.4 [42] and annotated using SnpEff v5.0e [43]. An augmented genome with both the U00096.2 reference genome and the integrated plasmid with the *tet* resistance mechanism (above) was used to determine transposon insertion sites within the plasmid. Briefly, sites containing discordant paired-end reads were collected whenever one end of the read aligned to the plasmid and one end aligned to the *E. coli* genome. Reads aligned to these collected sites were then tallied as either matching the genome reference or containing a breakpoint using a custom Python script available on our GitHub page.

### Genotyping of isolates

**Figure 3:**
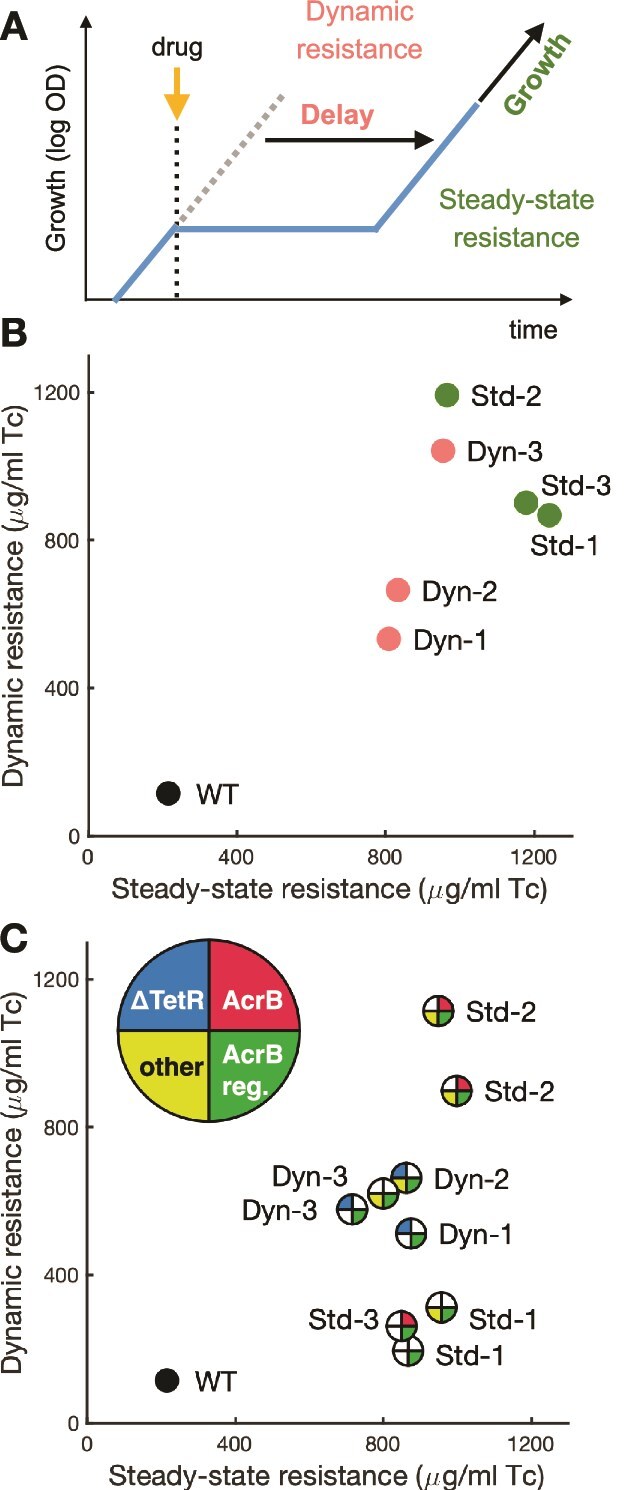
Mutations optimizing *acrB* provide the highest resistance and shortest recoveries. (A) In experiments where liquid cultures are exposed to a step increase in drug concentration, we calculate *dynamic resistance* to measure the population-level capacity for quickly recovering growth following exposure, using the delay in the time taken to reach one doubling that is introduced by the addition of drug during mid-log phase. We calculate *steady-state resistance* to measure the capacity for steady-state growth in the presence of drug, using the maximum growth rate reached after exposure*.* (B) Dynamic and steady-state resistances for the final evolved populations. Populations Dyn-1 and Dyn-2, where *tetR* transposon insertions were fixed, performed worse in both measures. (C) Dynamic and steady-state resistances for select isolates. *tetR* transposon mutants showed high dynamic resistance, but mutants with mutations optimizing AcrB for tetracycline efflux performed best.

**Figure 4:**
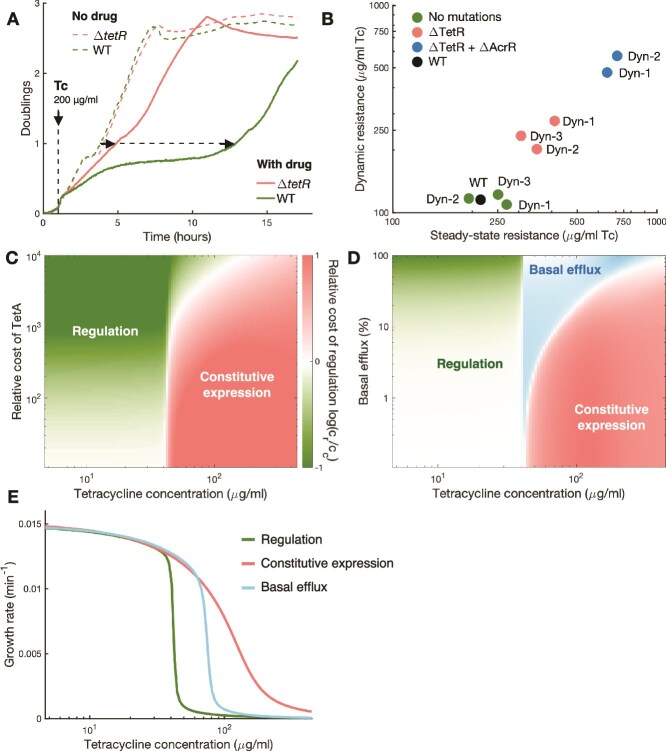
Sudden exposures to high drug doses overwhelm regulated responses. (A). Growth curves of isolates picked from the same population on the same day, one with a transposon insertion in the *tetR* gene and one without*.* The vertical dashed line indicates the addition of 200 μg/ml tetracycline. The corresponding growth curves under no drug are also included in dashed lines for comparison. The delays for each culture to reach one doubling following drug exposure are indicated by dashed arrows*.* Loss of TetR function reduces the recovery time significantly. (B) Dynamic and steady-state resistances for isolates with and without *tetR* transposon insertions, and also with further regulatory mutations in the *acr* operon. Loss of TetR function provides a significant increase in resistance, but further upregulation of the *acr* operon increases resistance even further. (C) We simulate drug responses using a mathematical model of the drug response. We simulated the system with and without TetR repression during exposures to a range of tetracycline concentrations. To compare the two regulatory strategies, we calculated costs associated with the decrease in cell growth and with the burden of unnecessary TetA production, considering scenarios where constitutive TetA expression has different relative contributions to the overall cost of the response. The color scale represents the relative cost of regulation $\log \left({c}_r/{c}_c\right)$, where ${c}_r$is the cost associated with the regulated response and ${c}_c$ is the cost of the constitutive response (Methods). Positive values indicate where constitutive expression is preferred, and negative values indicate where regulated expression is preferred. Regardless of the relative cost of TetA, constitutive expression is preferred during exposures to high drug doses. (D) We simulated the *tet* regulated response supplemented by a constant basal expression of the AcrB efflux pump. We simulated the system for different levels of basal AcrB efflux activity in comparison to the efflux achieved by fully induced TetA, and relative cost of TetA of 100 (Methods). Basal AcrB expression allows successful induction of the response at higher drug concentrations than the regulated response alone, and therefore it is preferred in a window of drug concentrations where it costs less than constitutive TetA expression. The shade of the color indicates the preference for each regulatory strategy, measured by the relative cost in relation to the second-best alternative. (E) Simulations showing final steady-state growth following exposures to different drug concentrations for regulated and constitutive expression of resistance, as well as regulated expression of TetA with the addition of a basal constitutive expression of AcrB corresponding to 10% of the efflux activity of fully induced TetA. There is a significant window of selection where constitutive expression of TetA allows for the maintenance of cell growth during drug exposures, although TetR-regulated TetA expression fails to induce a response. Basal expression of efflux pumps allows regulated resistance to successfully induce the response at higher drug concentrations.

The presence of selected SNPs in isolates was confirmed via Sanger sequencing. Glycerol stock samples for each population for each day were inoculated 1:100 in LB and grown for 2 h, then streaked on LB agar plates and grown at 37°C overnight. Ten individual colonies from each sample were randomly picked, selected genomic regions were PCR amplified and cleaned (Qiagen) and sent for sequencing to the Dartmouth College Genomics and Molecular Biology Shared Resources Core. Isolates known to have SNPs were preserved as glycerol stocks at −80°C. For samples with transposon insertions, the PCR product was assessed by gel electrophoresis to confirm insertions. In *tetR* mutants, we confirmed loss of TetR function by transformation with a plasmid expressing mCherry from the *tet* promoter, which results in high fluorescence levels in the absence of TetR repression. We selected a panel of isolates from the final evolved populations for further analysis in Fig. 3. To measure the effects of *tetR* insertions in Fig. 4, we picked isolates with and without relevant mutations from the first day when these mutations were detected to minimize the chance that these isolates also carried additional mutations.

### Plate reader experiments

Glycerol stock of relevant samples was inoculated into M63 or LB media and grown overnight. Each strain was inoculated 1:1000 in M63 and exposed to a gradient of 12 tetracycline concentrations (100–1000 μg/ml) during early log phase. Experiments were carried out at 37°C in a Synergy Neo2 plate reader (BioTek). We measured both the time until one doubling was achieved following exposure and the steady-state growth rate after recovery.

### Dynamic and steady-state resistances

From the plate reader experiments, we extract two complementary measures of resistance: (i) *Dynamic Resistance,* which is the drug concentration that introduces a 2-h delay in the time to reach one doubling following drug exposure in comparison to the absence of drug, and (ii) *Steady-State Resistance*, which is the drug concentration that halves the growth rate of the recovered population (similar to the IC_50_ concentration).

### Mathematical model

A full description of the model and its parameters is available in the SI, and a complete analysis is available elsewhere [44]. In short, we model the main biochemical interactions involved in the response as a set of differential equations that describe changes in the concentrations of the intracellular drug ($d$), the efflux pump TetA ($a$), and the repressor protein TetR ($r$):


$$ \dot{d}={K}_i\left(D(t)-{d}_f\right)-\frac{K_aa{d}_f}{k_a+{d}_f}-\lambda d $$

$$ \dot{a}=f\ {H}_a\left({r}_f\right)-\lambda a $$

$$ \dot{r}=f\ {H}_r\left({r}_f\right)-\lambda r $$

where ${K}_i$ stands for the import rate, D for extracellular drug concentration, ${d}_f$ for free intracellular drug, ${K}_a$ for the catalytic rate constant of TetA, ${k}_a$ the Michaelis constant, respectively,${r}_f$ free repressor, and ${H}_a\left({r}_f\right)$ and ${H}_r\left({r}_f\right)$ the synthesis rates for TetA and TetR that depend on free TetR. The terms $\lambda d$, $\lambda a$ and $\lambda r$ represent the dilution due to cell growth of drug, TetA and TetR, where $\lambda$ is the current growth rate. Both the growth rate $\lambda$ and $f$, which allow us to modulate the strength of gene expression according to the proteome partition, depend on nutrient levels and the intracellular drug concentration. To model these dependencies, we used the framework introduced by Scott et al. [45].

### Simulations

We numerically integrate the system of differential equations using the ode113 function in MATLAB, using values of external drug concentration and as an input parameter. The system starts in equilibrium in the absence of drugs, and then the external drug concentration is switched on. The parameter values used in the simulations are summarized in the SI and were either estimated from our experimental data [7], or obtained from literature [33]. To simulate loss of TetR function, we set ${H}_r\left({r}_f\right)=0$. To calculate the relative fitness of strains with WT regulation vs. constitutive TetA expression, we attributed costs to a decrease in the final steady-state growth rate following drug exposure ${c}_{\lambda }=1/{\lambda}_{final}$ and to the unnecessary expression of TetA in the absence of drugs ${c}_a={a}_0$ (in the absence of TetR regulation, TetA is fully expressed at all times). Because the relative fitness costs of decrease in resistance and TetA expression are hard to compare and highly depend on the environment (e.g. frequency and duration of exposures), we introduced a variable weight $w$ adjusting the cost of TetA expression, for a total cost of $c=w\ {c}_a+{c}_{\lambda }$. The fitness advantage of loss of TetR function was then calculated as the relative cost between constitutive TetA expression and WT regulation $\log \left({c}_{\Delta TetR}/{c}_{wt}\right)$. To simulate AcrB expression, we added a term for basal expression of the efflux pump, representing the contribution of AcrB to the total tetracycline efflux, without differentiating between AcrB and TetA. We considered the cost of AcrB to be proportional to the TetA concentration with equivalent efflux.

## Results

We have developed a device for microbial evolution in controlled dynamic environments, based on the morbidostat design [21], which propagates continuously growing cultures while automatically adjusting media input and antibiotic concentration to impose specific regimens of drug administration. The device allows the maintenance of cultures at a set density by measuring turbidity (optical density) in real-time and externally adjusting media and drug delivery via a control algorithm. We used our setup to impose both a “Steady” and a “Dynamic” environment to the evolving populations, implemented by different control algorithms.

### Steady environment favors mutations adapting AcrB to tetracycline

Most experimental evolution studies of antibiotic resistance have focused on the acquisition of resistance by sensitive strains, while less attention has been given to the evolution of strains already carrying dedicated mechanisms of resistance [46]. Previous evolution studies with a sensitive *E. coli* strain lacking the *tet* operon resulted in only a 10-fold increase resistance to tetracycline, with most adaptive mutations found in genes coding for membrane proteins, including the *acr* operon, or factors affecting transcription and translation [21]. For comparison, strains carrying the *tet* operon (*tet+*) achieve much higher resistance, with an MIC 100-fold larger than sensitive strains [7]. This raised the question of if the evolution of *tet+* strains would target the *tet* operon itself. If the native *tet* operon could be further optimized for higher doses of tetracycline, mutations in the *tet* genomic locus were likely to have large effects on resistance. Otherwise, if the *tet* operon were already highly optimized, adaptive mutations would be expected elsewhere, either providing resistance through other means or compensating for fitness costs associated with high TetA expression [47–49].

To determine the evolutionary trajectory of *tet*+ strains to increased tetracycline resistance, we evolved 3 populations (Std-1, Std-2, and Std-3) under a Steady regimen following the morbidostat design (Fig. 1B and C, Supplementary Fig. S1, Methods). In this regimen, the drug is always present, with levels gradually increasing as the population evolves resistance. Populations were sampled daily throughout the experiment. To measure the increase in drug resistance in each population, we inoculated a sample from each day in a gradient of tetracycline, measuring the drug concentration that reduces growth by half relative to growth in the absence of drug (IC_50_). By the third day, all three steady populations had developed high levels of resistance (Fig 1D and E, Supplementary Fig. S2). Next, we sent genomic DNA from each final culture for whole genome sequencing to identify adaptive mutations.

All three steady cultures evolved genetically diverse populations (Table 1) with significantly increased resistance in comparison to the wild type. We did not find any mutations in the *tet* operon locus, suggesting that TetA is already highly optimized for tetracycline efflux. Instead, we observed a predominance of mutations enhancing other mechanisms of resistance, particularly the *acr* operon. All three populations acquired mutations upregulating AcrB, either by disrupting repressor AcrR or the *acrRAB* promoter region. Additionally, two populations acquired mutations in the drug binding pocket of the AcrB protein itself [37, 38], presumably increasing the affinity of AcrB to tetracycline (Fig. 1F). Therefore, in Steady populations resistance is increased by optimizing the *acr* operon for tetracycline efflux, complementing the resistance provided by TetA.

**Table 1:** Relevant mutations found in evolved Steady populations. Mutations shown are present above a 0.15 frequency and are not detected in the ancestral WT population.

Population	Gene	Function	Frequency
Std-1	*p-acrRAB::IS2*	Multidrug efflux pump	0.86
Std-1	*plsB*	Phospholipid biosynthesis, multidrug tolerance	0.63
Std-1	*insB1*	Insertion element IS1 protein	0.15
Std-1	*del cheA/* *motAB/flhCD*^a^	Chemotaxis, motility	1.00
Std-2	*p-acrRAB*	Multidrug efflux pump promoter	0.99
Std-2	*ompR* (R15S)	Porin regulator, osmotic stress	0.97
Std-2	*yhdP*	Phospholipid transport factor	0.37
Std-2	*acrB* (Q284H)	Multidrug efflux pump	0.36
Std-3	*acrR::IS1*	AcrB multidrug efflux pump repressor	0.78
Std-3	*acrB* (F281I)^a^	Multidrug efflux pump	0.75
Std-3	*p-rrsG* (2729370)	16S ribosomal RNA	0.27
Std-3	*p-rrsG* (2729333)	16S ribosomal RNA	0.24
Std-3	*del cheA/* *motAB/flhCD*^a^	Chemotaxis, motility	1.00

a Found in very low abundance in the ancestral WT population*.*

In addition to mutations in the *acr* operon, we also observed other mutations that potentially affect membrane permeability to tetracycline, such as in porin regulator OmpR, or in PlsB and YhdP, which are involved in phospholipid synthesis and transport [50, 51]. In all evolved populations, there were several low-abundance mutations related to transposable elements (discussed below). Population Std-3 showed two mutations upstream of 16S rRNA gene *rrsG*, which codes for the target of tetracycline and is possibly upregulated in response to the drug. Additionally, a large deletion of chemotaxis and motility genes that were already detected in low abundance in the ancestral WT population became fixed in populations Std-1 and Std-3. Although these genes are not necessary for growth in chemostat conditions, and therefore their deletion might promote fast growth regardless of the presence of antibiotics, we speculate that the deletion of several motor proteins involved in motility might also alleviate the depletion of the membrane potential caused by constant TetA expression.

To establish the order in which mutations arose, we analyzed the emergence of the most abundant mutations in the evolved populations: *plsB* (Std-1), *acrB* (Std-2 and Std-3) and *ompR* (Std-2 and Std-3). We picked five isolates randomly from each population for each day of the experiment and verified the presence of mutations in each gene by Sanger sequencing. We then determined the order and co-occurrence of mutations to infer the competing lineages in each population over the course of the experiment (Fig. 1G). In addition to the expected mutations, we also found several other competing mutations in the *acr* operon that were not detected in the final populations. Mutations in the *acrB* gene itself were always preceded by mutations disrupting AcrB repression, suggesting that tetracycline is not an effective inducer of the *acr* operon, and upregulation of *acrAB* is necessary to potentialize further mutations in *acrB*. In particular, population Std-1achieved upregulation of *acrAB* through an *IS2* transposon insertion within its promoter region, with no further mutations anywhere in the *acr* operon. The *IS2* insertion sequence is known to introduce a strong promoter that results in upregulation of adjacent genes [52]. Overall, these results indicate that the evolution of nonoptimal resistance mechanisms in steady drug regimens proceeds first by upregulation of the resistance protein, followed by the optimization of the resistance proteins themselves.

### Dynamic environment favors loss of *tet* regulation

Experimental evolution studies of antibiotic resistance have typically been conducted under steadily increasing antibiotic concentrations, which does not account for the dynamic nature of most natural habitats such as clinical settings [53, 54]. In these environments, although drug exposures can be relatively rare, they happen suddenly and at high concentrations, requiring regulated mechanisms of resistance to deliver fast responses. Therefore, evolution in such highly dynamic environments should be expected to lead to faster induction of resistance. However, previous studies have shown that drastic changes in the environment impose strict evolutionary bottlenecks that limit the availability of evolutionary paths towards optimized phenotypes [23]. The evolution of faster responses might then depend on rare mutations that are not likely to occur under the bottlenecks imposed by sudden exposures to high drug doses. Therefore, it was unclear if faster regulation of the *tet* operon would be a likely outcome of evolution in such environments.

To determine the role of dynamics in the evolution of drug responses, we evolved three resistant populations (Dyn-1, Dyn-2, and Dyn-3) under a Dynamic drug environment, implemented by a daily single large dose of tetracycline followed by growth in fresh medium (Fig. 2A–C, Supplementary Fig. S1, Methods). By adjusting the drug dose based on the previous recovery time of the population, we maintain the evolutionary pressure for faster response times, while the subsequent growth in fresh medium provides a fitness advantage for cells that grow efficiently in the absence of drug. A regimen of daily administration of the drug was chosen to provide enough time for growth in drug-free conditions and to reflect typical timescales of antibiotic treatments. Next, we measured the increase in drug resistance in each population throughout the experiment. Overall, all three Dynamic populations increased resistance more slowly in comparison to the Steady populations, as measured by the IC_50_ (Fig 2D and E, Supplementary Fig. S2). These results support the notion that strict evolutionary bottlenecks imposed by the Dynamic environment can select mutants that are less fit in comparison to the Steady environment.

The Dynamic regimen produced even higher genetic diversity than the Steady regimen (Table 2). However, instead of evolving fast on/off switching of the drug response, all Dynamic populations independently acquired *IS5* transposon insertions in different loci inside the *tetR* gene, resulting in loss of TetR function. We verified the presence of these insertions in each population throughout the experiment by PCR and found that *IS5* insertions in *tetR* rapidly emerged and reached fixation in populations Dyn-1 and Dyn-2 (Fig. 2F, Supplementary Fig. S3). In population Dyn-3, *tetR* insertion only rose in frequency in the last few days but did not reach fixation. Such insertions abolish regulation of efflux pump TetA, which becomes permanently de-repressed. Nonetheless, TetA levels are expected to be similar between *tetR* mutants and WT cells when fully induced in the presence of the drug. These results suggest that constitutive expression of TetA improves resistance by bypassing the induction of the response, and therefore we hypothesized that preemptive expression of resistance could shorten population-level recovery to sudden drug exposures despite the known fitness costs of TetA overexpression.

**Table 2:** Relevant mutations found in evolved Dynamic populations. Mutations shown are present above a 0.15 frequency and are not detected in the ancestral WT population.

Population	Gene	Function	Frequency
Dyn-1	*tetR::IS5* (1)	TetA tetracycline efflux pump repressor	0.97
Dyn-1	*acrR::IS1*	AcrB multidrug efflux pump repressor	0.73
Dyn-1	*del cheA/* *motAB/flhCD*^a^	Chemotaxis, motility	1.00
Dyn-2	*tetR::IS5* (2)	TetA tetracycline efflux pump repressor	0.99
Dyn-2	*acrR::IS1*	AcrB multidrug efflux pump repressor	0.66
Dyn-2	*rpoB*	RNA polymerase subunit β	0.52
Dyn-2	*kgtP*	Transporter for α-ketoglutarate	0.20
Dyn-2	*rhsA*	Rearrangement hotspot element	0.18
Dyn-2	*insF1*	Insertion element IS3 transposase	0.15
Dyn-2	*rrfF*	5S ribosomal RNA	0.15
Dyn-2	*del tar/cheAW/motAB/flhCD* ^a^	Chemotaxis, motility	1.00
Dyn-3	*ompR* (R15S)	Porin regulator, osmotic stress	0.67
Dyn-3	*p-acrRAB*	Multidrug efflux pump promoter	0.61
Dyn-3	*intK*	Prophage, drug resistance	0.30
Dyn-3	*tetR::IS5* (3)	TetA tetracycline efflux pump repressor	0.27
Dyn-3	*flu*	Prophage, self-recognizing antigen	0.23
Dyn-3	*acrR* (S31Y)	AcrB multidrug efflux pump repressor	0.23
Dyn-3	*rhsB*	Rearrangement hotspot element	0.20
Dyn-3	*insL1*	Insertion element IS186 transposase	0.19
Dyn-3	*trmA*	tRNA methyltransferase	0.15

a Found in very low abundance in the ancestral WT population.

In all Dynamic populations, we also observed a high abundance of *acr* regulatory mutations. However, we did not find any mutations in the *acrB* gene. Populations Dyn-1 and Dyn-2 both showed *IS1* transposon insertions in repressor *acrR*, and population Dyn-3 showed both mutations in the *acrRAB* promoter and in *acrR* itself. Therefore, upregulation of AcrB also increases fitness in the Dynamic environment. Again, we observed other prevalent mutations that potentially reduce membrane permeability to tetracycline or compensate for its effects, such as *ompR,* RNA polymerase subunit *rpoB* and the *kgtP* transporter, as well as several mutations related to transposable elements. In populations Dyn-1 and Dyn-2, we also found the fixation of the large deletion of chemotaxis and motility genes that might help compensate for TetA overexpression.

To establish the order in which mutations arose, we picked five isolates randomly from each population for each day of the experiment and verified the presence of the most abundant mutations: *acrR* (Dyn-1, Dyn-2, and Dyn-3), *rpoB* (Dyn-2) and *ompR* (Dyn-3). We then determined co-occurrence of mutations within the same lineages to infer the competing lineages in each population (Fig. 2G). In all populations, *tetR* insertions preceded any other mutations and were inevitably followed by mutations upregulating the *acr* operon. However, while *tetR* insertions were quickly fixed in populations Dyn-1 and Dyn-2, population Dyn-3 showed an interesting case of clonal interference. Although a *tetR* insertion was detected early, by day four it was competing with another lineage carrying mutations in *ompR* and in the *acRAB* promoter. The lineage with the *tetR* insertion then developed two competing *acr* regulatory mutations before rising to ~30% abundance in the last 4 days of the experiment. Therefore, mutations outside of the *tet* operon can also significantly increase fitness in dynamic environments and interfere with the emergence of *tetR* mutants. Next, we moved to study the dynamics of the response in the evolved populations and in relevant isolates.

### Mutations optimizing *acrB* provide highest resistance and shortest recoveries

To measure antibiotic resistance in the evolved populations during abrupt changes in drug concentration, we developed a liquid-culture assay based on the dynamics of population-level growth during drug responses [55, 56] (Fig. 3A). We grow cultures in the absence of drugs until mid-exponential phase, and then suddenly expose the cultures to large doses of tetracycline, picked from a gradient, which is then sustained for the rest of the experiment. Sudden exposures to high drug concentrations cause the growth of liquid cultures to pause temporarily, as a large subpopulation of cells is arrested [7]. Growth is then resumed when the growing subpopulation of surviving cells surpasses this background of arrested cells. We then quantify resistance using two components: “steady-state resistance,” which measures the capacity for bulk growth in the presence of drug, and “dynamic resistance,” which measures the speed of population-level recovery following drug exposure. Both the speed of recovery and the ensuing steady-state growth rate decline as a function of the drug concentration used in the exposure. Therefore, we define steady-state resistance as the drug concentration that reduces the steady-state growth rate of the recovered population to half of drug-free growth (similar to IC_50_), and we define dynamic resistance as the drug concentration that introduces a 2-h delay in the time it takes the population to achieve one doubling following drug exposure, in comparison to growth in the absence of drug (methods, Supplementary Figs S4–S7).

Although all populations evolved similar levels of steady-state resistance, we found significant differences in dynamic resistance. Despite the frequent sudden exposures to tetracycline, populations evolved in the Dynamic regimen showed lower dynamic resistance than the populations evolved in the Steady regimen (Fig. 3B, Supplementary Fig. S5). Therefore, mutations acquired under constant drug pressure can also shorten population recovery following sudden drug exposures. Evolved populations even grow slightly faster in the presence of moderate concentrations of tetracycline than in its absence (Supplementary Fig. S5). Because the evolved populations harbor several mutants, we also assessed resistance in a collection of representative isolates (Fig. 3C, Supplementary Table S2, Supplementary Fig. S6). We found that although all isolates from the Dynamic regimen showed increased dynamic resistance compared to the WT, the highest dynamic resistance was measured in isolates from Steady population Std-2 combining mutations in *acrB* and *ompR.* Therefore, mutations improving the efficiency of tetracycline efflux are likely to result in both faster recovery from drug exposures and faster steady-state growth at high drug concentrations. In contrast, regulatory mutations that shorten recovery from exposure, such as *tetR* transposon insertions, do not necessarily improve steady-state growth at high drug levels.

### Sudden exposures to high drug doses overwhelm regulated responses

To understand the fitness advantage provided by loss of *tetR* repression, we picked isolates from the same sample of early Dynamic populations where *tetR* insertions first emerged. We then compared how isolates with and without *tetR* insertions respond to sudden exposures to tetracycline (Fig. 4A). Liquid cultures of isolates with *tetR* insertions recovered growth much earlier following exposure, despite little change in the steady-state growth rate once growth was recovered. Therefore, abolishing TetR repression of TetA increases cell survival to the initial exposure and recovery of population growth, but steady-state growth under full TetA induction is still similar between WT and *tetR* mutants.

Once *tetR* mutations are acquired, upregulation of *acrB* provides further increase of both recovery speed and steady-state growth. We measured dynamic and steady-state resistance in WT, *tetR* mutants and in *tetR + acrR* mutants (Fig. 4B, Supplementary Fig. S7). *tetR* insertions increased dynamic resistance substantially, while providing only a slight increase in steady-state resistance. Therefore, because WT and *tetR* mutants showed similar steady-state growth rates for all drug concentrations where they are both viable (Supplementary Fig. S7), the selective advantage of *tetR* mutants likely results from WT cells not being able to fully induce TetA expression in time. In contrast, further acquisition of *acrR* mutations by *tetR* mutants resulted in much higher gains in both steady-state and dynamic resistance, corroborating the hypothesis that mutations that improve tetracycline efflux increase resistance in any regimen of drug administration.

Regulatory mutations are more evolutionarily accessible and likely to precede coding mutations, but targeted coding mutations optimizing resistance proteins provide higher gains in resistance. Tetracycline efflux provided by AcrB is in addition to the efflux provided by TetA, increasing the total efficiency of tetracycline efflux. Mutations increasing AcrB tetracycline efflux then provide both faster steady-state growth and faster recovery from drug exposures, increasing steady-state and dynamic resistances alike. Specific coding mutations in *acrB*, which are observed in the Std-2 mutants (Fig. 3C), likely increase AcrB affinity for tetracycline and impact drug efflux more than regulatory mutations alone, resulting in higher resistance by both counts. In contrast, *tetR* transposon insertions only shorten recovery from drug exposures without improving the efficiency of tetracycline efflux, providing only modest gains in dynamic resistance.

To understand how sudden drug exposures can select for loss of TetR repression, we developed a mathematical model of the dynamics of drug responses [44, 57, 58] that tracks the accumulation of intracellular tetracycline and expression of both TetA and TetR. This model includes drug effects on growth and gene expression [45, 59], and loss of TetR repression is modeled by simply reducing TetR expression to zero (Methods, SI). For both the presence and absence of TetR regulation, we simulated the model in a range of external tetracycline concentrations (Fig. 4C–E). We then compared the two regulatory strategies by calculating costs associated with the decrease in cell growth and the burden of unnecessary TetA production in each case. Because the fitness cost of TetA is difficult to determine, we considered different values for the relative contribution of TetA expression to the overall cost of the response. Calculating the overall cost of the drug response for each regulatory strategy, we found that, regardless of the burden associated with TetA expression, regulated TetA expression is optimal during exposures to lower drug concentrations and constitutive TetA expression is optimal at higher drug concentrations (Fig. 4C). Therefore, because exposure to high drug concentrations can result in drug influx outpacing expression of resistance, constitutive expression is preferred despite its potential costs.

To understand the role of *acr* mutations in tetracycline resistance, we introduced a basal level of AcrB expression to the model in addition to the *tet* regulated response. Pre-existing levels of resistance reduce the initial influx of drug into the cell upon sudden exposures, buying more time for TetA expression and increasing the likelihood of a successful response. Moreover, additional levels of resistance in the steady state provided by AcrB allow growth at higher drug concentrations than fully induced or constitutive TetA expression alone. Simulating the system for different levels of basal AcrB efflux, we find that even relatively low AcrB drug efflux extends the range of drug concentrations for which regulated resistance can induce a successful response (Fig. 4D and E). This explains why mutations upregulating AcrB produce higher dynamic resistance, in addition to higher steady-state resistance. For high AcrB drug efflux, not only the cell survives sudden exposures to high drug concentrations, but it also grows at higher drug levels than what is achievable by TetA alone. Therefore, high AcrB efflux in addition to TetA is the preferred mechanism for exposures to high tetracycline concentrations, which explains why lineages with coding mutations in *acrB* achieved the highest levels of resistance by any measure.

### Mobile elements as a general strategy of mutating regulatory pathways

Both the Steady and Dynamic environments resulted in an abundance of regulatory mutations, and many of these were implemented by the insertion of transposable elements. Crucially, all Dynamic populations evolved independent *IS5* transposon insertions inside the *tetR* gene, making TetA expression constitutive. All six populations from both environments evolved upregulation of *acrB*, and four of these mutations were also mediated by transposon insertions. Overall, the highest frequency mutations included several examples of disrupted transcriptional repressors and their binding sites, with about half of these regulatory mutations mediated by transposon insertions. Apart from disrupting regulation, transposon insertions also introduced an additional promoter for *acrB* and mediated the large deletion of chemotaxis and motility genes. Populations in both environments also exhibited a wealth of low-frequency mutations in genes related to mobile genetic elements [60, 61] (Tables 1 and 2). Comparatively fewer mutations were observed in non-regulatory genes (*acrB*, *rpoB*, and *plsB*), and these mutations only appeared later in the experiment in lineages that already possessed regulatory mutations. Thus, these results support the notion that microbial evolution in new environments proceeds first through regulatory mutations, which are more accessible than the low probability point mutations necessary to reach optimal phenotypes. Mobile genetic elements then provide a fast mechanism for upregulating useful genes, either by disrupting repressors or inserting new promoters, or also deleting harmful genes.

## Discussion

Performing experimental evolution using carefully controlled continuous cultures, we studied the role of the dynamics of drug delivery in the evolution of antibiotic responses. We found that steady environments where drug concentrations change only gradually led to the refinement of the AcrB efflux pump through point mutations that optimize it for tetracycline efflux. In contrast, dynamic environments with sudden exposures to large drug concentrations did not result in such refinement of protein function, but instead led to the abolishment of regulation of the *tet* operon, the main mechanism of tetracycline resistance. Such mutation was unexpected in a changing environment, because loss of regulation had been previously understood to be a long-term outcome of evolution in constant environments [62]. These experiments show that regulation is not only important for the parsimonious use of cellular programs that are only needed occasionally but also to guarantee a timely activation of cell defenses in hostile environments, while gene expression is still possible. We speculate that the strict evolutionary bottlenecks imposed by sudden drug exposures diminish the probability of occurrence of the relatively rare mutations refining AcrB for tetracycline efflux.

Regardless of environment, evolution proceeded first through regulatory mutations [63–65], with a high prevalence of transposon insertions. Previous work shows that exposure to low levels of tetracycline results in high levels of mobile element activity via the sensing of cellular stress [66–68]. Evolution under high drug levels requires quick adaptation, and transposon insertions provide an accessible mechanism for genome remodeling. Transposon insertions also offer the advantage of being reversible [69]. The overwhelming presence of transposon insertions rewiring the regulation of resistance genes, as well as mutations in genes regulating and operating transposable elements, indicates an important role of this mechanism in the short-term evolution of microbes in challenging environments, which is often missed in whole-genome sequencing analysis.

Sudden drug exposures are likely to play a role in the evolution of pathogens in clinical environments. Most antibiotic treatments are well designed to reach and maintain high drug concentrations, but short courses such as intravenous or inhalation result in sharp increases of drug bioavailability at the site of infections [70]. In particular, although tetracycline administered orally typically reaches systemic levels of ~5 μg/ml [28], which is below the concentrations used in this study, topical administration can quickly reach much higher levels [71]. As a result, constitutive expression of resistance genes is often found in clinical environments. For example, a high proportion of plasmid-born resistance mechanisms is not regulated [72]. Although opportunistic pathogens typically regulate their chromosomally encoded resistance mechanisms [73], loss-of-function mutations in transcription repressors of resistance genes are often found in clinical isolates [74, 75]. Our results suggest that these mutations can serve both the purposes of bypassing the induction of drug responses and of de-repressing resistance mechanisms that are not sufficiently activated by the drug. Constitutive expression of resistance would be even more beneficial for resistance mechanisms that are induced only by downstream effects of the drug and respond much more slowly than the *tet* operon [76]. Overall, our work helps explain the evolution of constitutive expression of resistance by identifying its benefits in highly variable environments, which might help offset its costs. Additionally, the induction of resistance mechanisms is also known to be important for persistence, the recovery of growth after the antibiotic challenge has subsided [77, 78]. Future studies will determine if the regulatory mutations we found here are also beneficial for microbial cells that are unable to recover growth in the presence of drug.

This work raises interesting questions about how the dynamics of the environment shapes evolution, such as whether dynamic regimens with lower drug concentrations might ease evolutionary bottlenecks and ultimately select for faster responses, or whether longer periods in the absence of drug might reduce the benefits of constitutive expression of resistance. Antibiotics with different modes of action or alternative mechanisms of resistance could also change population dynamics in dynamic environments. For instance, the use of bactericidal drugs can lyse most of the cell population and result in stricter evolutionary bottlenecks, or enzymes that inactivate the antibiotic can help reduce the drug concentration in the medium. Dynamic environments could also promote the coexistence of multiple mutants or species that are optimized for different drug levels, shaping the composition and evolution of complex microbial communities [79].

## Supplementary Material

SI_wraf082

calls_snp_wraf082

insertion_tallies_wraf082

isolates_wraf082

## Data Availability

The data that support the findings of this study are openly available. All data and code generated in the study are included in the SI and at our GitHub page: https://github.com/schultz-lab/Evolution-Dynamics.
