# Supplementary material for: Drug delivery dynamics dictate evolution of bacterial antibiotic responses
Source: ISME J. 2025 May 11;19(1):wraf082. doi: 10.1093/ismejo/wraf082 (PMC12086408; doi:10.1093/ismejo/wraf082)
Supplement: SI_wraf082 [file si_wraf082.pdf]

Supplemental Information

Drug delivery dynamics dictate evolution of bacterial antibiotic responses.

John C. Crow[1], Hao Geng[1], Christopher J. Geiger[1], Timothy J. Sullivan[2], Shannon M. Soucy[2], Daniel Schultz[1]

[1] Department of Microbiology & Immunology, Dartmouth – Geisel School of Medicine, Hanover, NH, USA.

[2] Department of Biomedical Data Sciences, Dartmouth – Geisel School of Medicine, Hanover, NH, USA.

Mathematical Model.

A complete analysis of this model is published in Stevanovic et al[53]. We model the main biochemical interactions involved in the response as a set of differential equations that describe changes in the concentrations of the intracellular drug (d), the efflux pump TetA (a), and the repressor protein TetR (r):

$$\dot{d} = K_i\left(D(t) - d_f\right) - \frac{K_a a d_f}{k_a + d_f} - \lambda d$$

$$\dot{a} = f\, H_a\left(r_f\right) - \lambda a$$

$$\dot{r} = f\, H_r\left(r_f\right) - \lambda r$$

where K_i stands for the import rate, D for extracellular drug concentration, d_f for free intracellular drug, K_a for the catalytic rate constant of TetA, k_a the Michaelis constant, respectively, r_f free repressor, and $H_a(r_f)$ and $H_r(r_f)$ the synthesis rates for TetA and TetR that depend on free TetR. To simulate loss of TetR function, we set $H_r(r_f) = 0$. Because the biochemical binding and unbinding of the substrate to the transcription factor typically happens at a much faster rate than the aforementioned processes, we consider their unbound (free) forms (d_f, r_f) to be in chemical equilibrium with the bound form $[rd]$ with a dissociation constant K_d, such that $r_f d_f = [rd]$. The term $K_i(D(t) - d_f)$ represents the diffusion of drug across the membrane into the cell, $\frac{K_a a d_f}{(k_a + d_f)}$ the export of drug out of the cell by the efflux pump. The terms λd, λa and λr represent the dilution due to cell growth of drug, TetA and TetR, respectively, where λ is the current growth rate. Both f, which allows us to modulate the strength of gene expression, and the growth rate λ depend on the nutrient level and the intracellular drug concentration.

To model these dependencies, we used the framework introduced by Scott et al[54]. In this framework κ_n and κ_t are the nutritional and translational capacity of the cell, respectively. Here, we refer to their base values (full nutrients, no drug) as κ_n^0 and κ_t^0, respectively.

Because cultures are kept at lower densities, in regimes where growth is not yet limited by nutrient depletion, we assume the nutritional capacity to remain at $\kappa_n = \kappa_n^0$. For the translational capacity, we assume inhibition by tetracycline according to $\kappa_t = \kappa_t^0 \frac{K_{ribo}}{K_{ribo} + d_f}$, where the fraction represents the fraction of free ribosomes in the cell, and K_{ribo} is the

dissociation constant for tetracycline and the ribosome. Lower K_{ribo} values correspond to stronger inhibition, and vice versa. The growth rate can then be calculated as $\lambda = \frac{\phi_c}{\rho} \cdot \frac{\kappa_t \kappa_n}{\kappa_t + \kappa_n}$, where $\phi_c = 0.48$ and $\rho = 0.76$ are universal constants.

κ_t^0 has a universal value of $0.075\, min^{-1}$ $\kappa_n$for *E. coli*. κ_n^0 varies according to nutrient quality. We chose $\kappa_n^0 = 0.035\, min^{-1}$ such that $\lambda_0 = \frac{\phi_c}{\rho} \cdot \frac{\kappa_t^0 \kappa_n^0}{\kappa_t^0 + \kappa_n^0}$ (corresponding to a state without tetracycline) yields a maximum growth rate of $\lambda_0 = 0.015\, min^{-1}$ as observed in our experiments with M63 medium.

We assume that TetA and TetR are part of the P sector of the proteome, which scales as $\phi_P = \phi_c \cdot \frac{\kappa_t}{\kappa_t + \kappa_n}$. Without regulation of the synthesis rates $H_a(r_f)$ and $H_r(r_f)$, we would expect the concentrations of TetA and TetR to scale in the same way. Hence, we define $f = \frac{\lambda}{\lambda_0} \cdot \frac{\kappa_t}{\kappa_t + \kappa_n} / \frac{\kappa_t^0}{\kappa_t^0 + \kappa_n^0}$, which leads to steady states proportional to $\sim \frac{\kappa_t}{\kappa_t + \kappa_n}$ and simplifies to $f = 1$ at full nutrients and no drug.

Parameters were either obtained from literature[33] or estimated from experiments[7].

Model parameters.

Parameter	Value	Definition	Parameter	Value	Definition
K_i	$0.015\ min^{-1}$	drug diffusion influx	$D(t)$	varies	extracellular drug
K_a	$50\ min^{-1}$	catalytic rate TetA	k_a	$10\ \mu M$	Michaelis constant TetA
K_d	$0.001\ \mu M$	TetR/Tc dissoc. constant	K_{ribo}	$1\ \mu M$	Tc/ribo dissoc. constant
$H_a(r_f)$	$A\frac{r_{0,a}^4}{r_{0,a}^4 + r_f^4}$	TetA synthesis regulation	$H_r(r_f)$	$R\frac{r_{0,r}^4}{r_{0,r}^4 + r_f^4}$	TetR synthesis regulation
A	$0.008\ \mu M\ min^{-1}$	maximal TetA synthesis	R	$0.0003\ \mu M\ min^{-1}$	maximal TetR synthesis
r_{0a}	$0.0001\ \mu M$	dissoc. constant TetA regulation	r_{0r}	$0.000075\ \mu M$	dissoc. constant TetR regulation
ϕ_c	0.48	proteome partition const.	ρ	0.76	conversion constant
κ_t^0	$0.075\ min^{-1}$	translational capacity	κ_n^0	$0.035\ min^{-1}$	nutritional capacity
λ_0	$0.015\ min^{-1}$	max. growth in M63 medium			

Table S1. Definitions and values of parameters used in the mathematical model.

Population	Gene	Function
Std-1	*p-acrRAB::IS2*	Multidrug efflux
Std-1	*p-acrRAB::IS2* + *plsB*	Multidrug efflux, phospholipid biosynthesis
Std-2	*p-acrRAB* + *ompR* + *acrB* (Q284H)	Multidrug efflux, porin regulator
Std-2	*p-acrRAB* + *ompR* + *acrB* (A286V)	Multidrug efflux, porin regulator
Std-3	*acrR::IS1* + *acrB* (F281I)	Multidrug efflux, AcrB repressor
Dyn-1	*tetR::IS5* + *acrR::IS1*	TetA repressor, AcrB repressor
Dyn-2	*tetR::IS5* + *acrR::IS1* + *rpoB*	TetA and AcrB repressors, RNAP subunit β
Dyn-3	*tetR::IS5* + *acrR* (S31Y)	TetA repressor, AcrB repressor
Dyn-3	*p-acrRAB* + *ompR*	Multidrug efflux, porin regulator

Table S2: Isolates assessed in Figure 3C. These isolates were picked from final evolved populations and represent the main mutations obtained in this study.

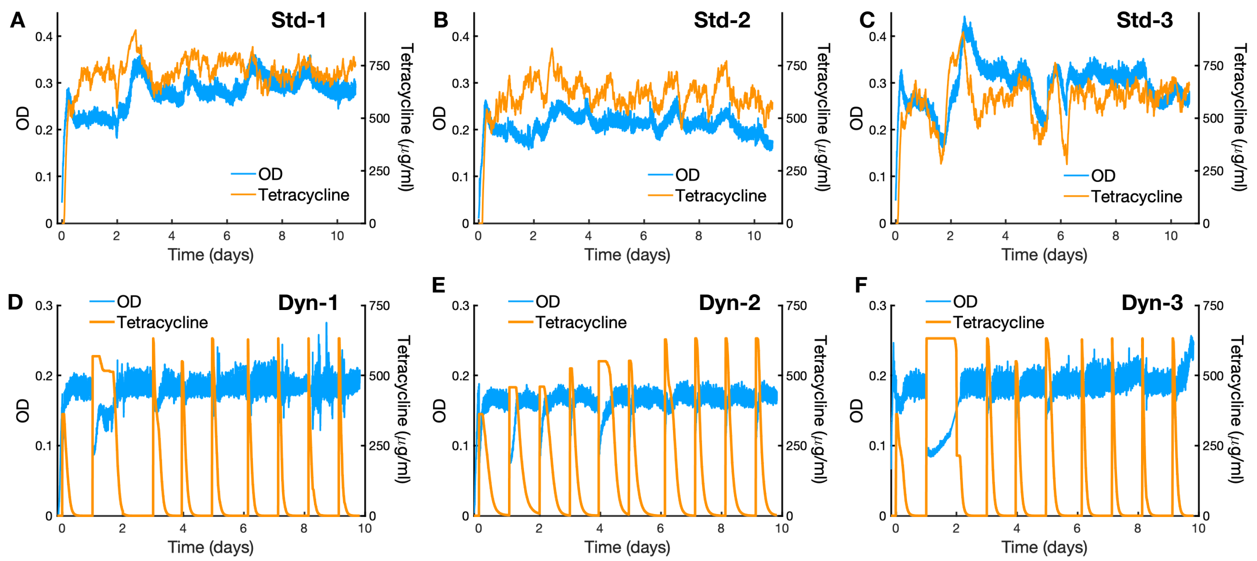

Figure S1. OD and tetracycline concentration over the course of the experiment. Optical densities and tetracycline concentrations measured over time for **A-C** Steady populations and **D-F** Dynamic populations. Populations Dyn-1 and Dyn-3 recovered slowly from the second exposure and skipped the third exposure.

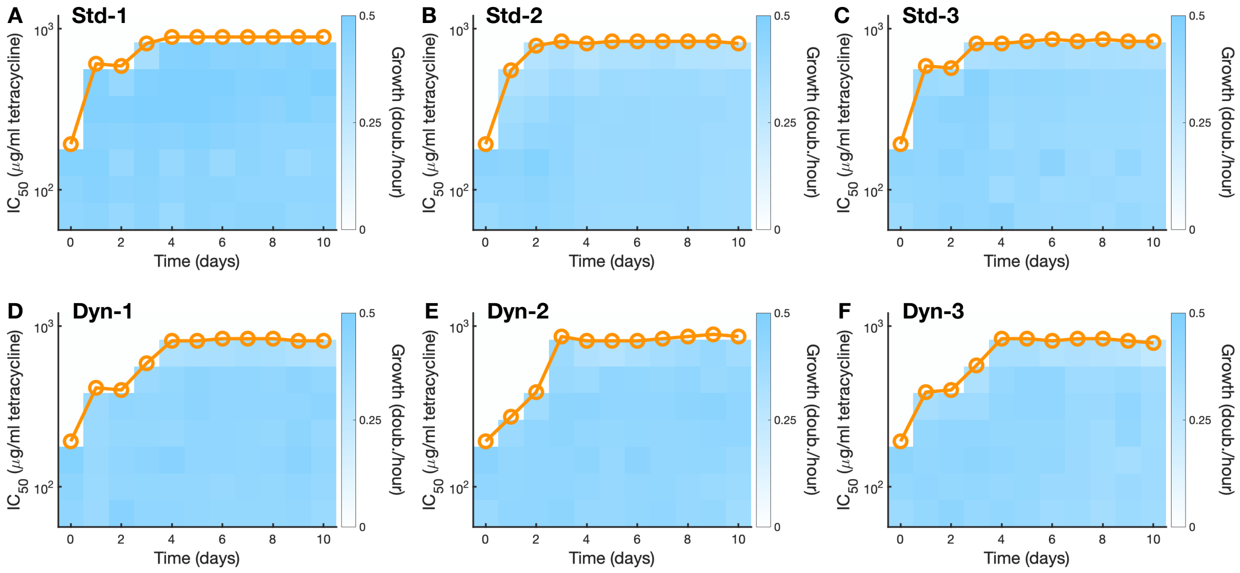

Figure S2. Progression of tetracycline resistance in each population. A-F. Samples from each population in each day were grown in tetracycline concentrations picked from a gradient and growth rates were determined in each case. Circles indicate the drug concentration at which growth is reduced to half (IC_{50} concentration).

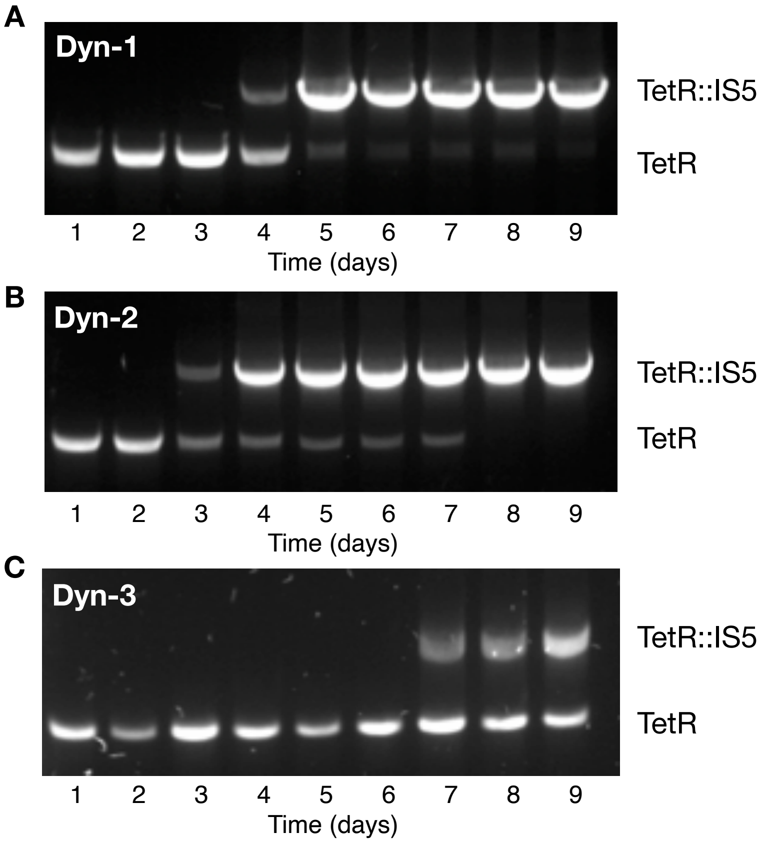

Figure S3. Prevalence of *IS5* insertions in the *tetR* gene over the course of the experiment. PCR amplification of the *tet* operon showing acquisition of transposon insertion in each Dynamic population. We quantified the relative intensity of the bands to estimate the prevalence of TetR transposon insertions in each population in each day.

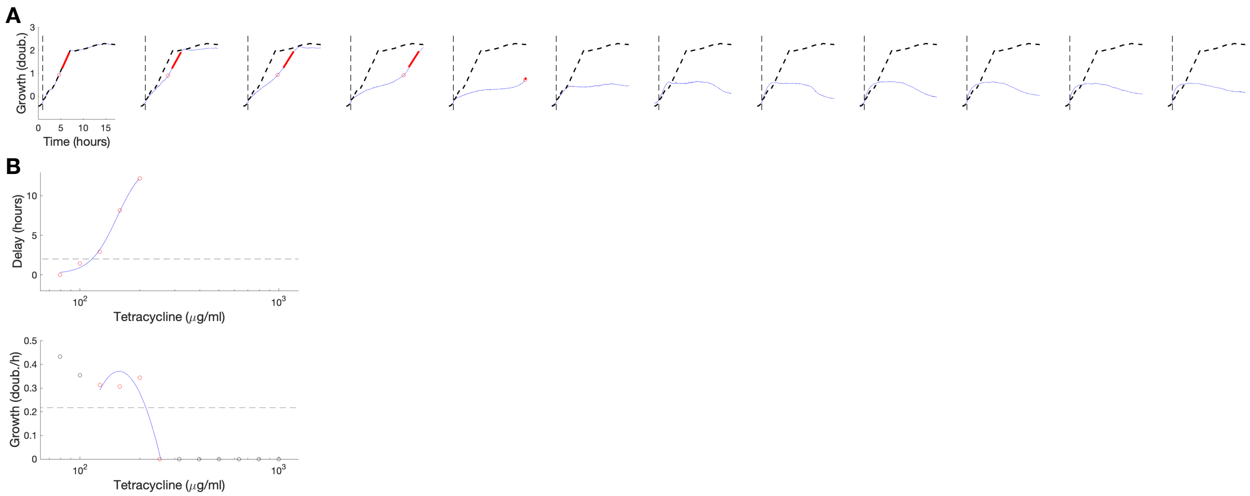

Figure S4. Calculation of steady-state and dynamic resistances for the WT ancestral strain. A. Growth curves of the WT ancestral strain. Plots are log_2(OD) over 20 hours. Vertical dashed line indicates addition of tetracycline picked from a gradient of zero in the first column then 100 to 1000 μg/ml in columns 2 to 12. The corresponding growth curve with zero drug is included in a dashed line for comparison. The point at which the culture reaches a doubling is indicated, as well as the linear fit during steady-state growth following recovery. **B.** Dynamic resistance is calculated as the drug concentration that causes a 2-hour delay in the time to reach one doubling following drug exposure. Steady-state resistance is calculated as the drug concentration that reduces steady-state growth to half of the growth rate under no drug. Curves were fit with second order polynomials.

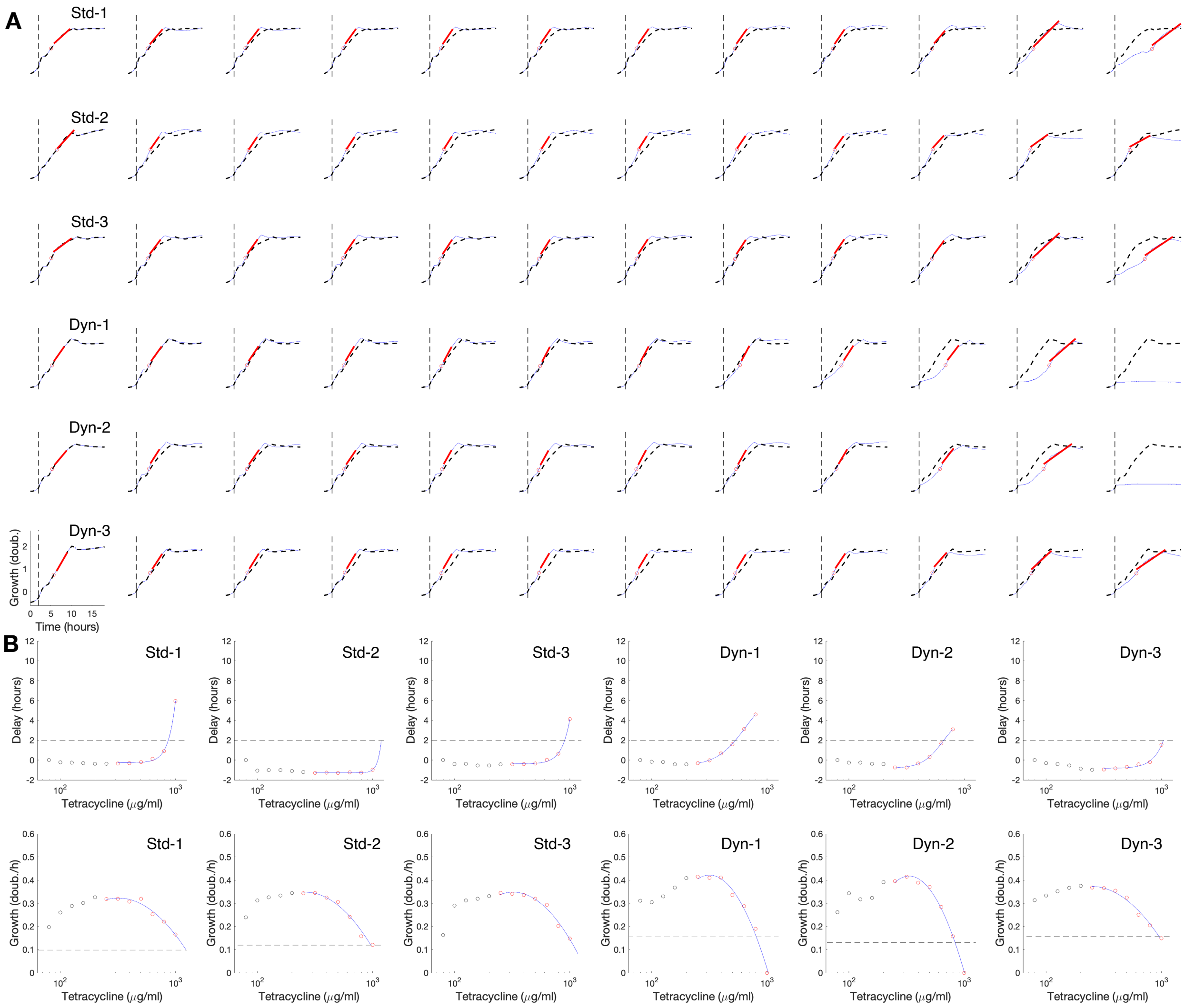

Figure S5. Calculation of steady-state and dynamic resistances for final evolved populations. A. Growth curves of the final populations. Plots are $\log_2$(OD) over 20 hours. Vertical dashed line indicates addition of tetracycline picked from a gradient of zero in the first column then 100 to 1000 μg/ml in columns 2 to 12. The corresponding growth curve with zero drug is included in a dashed line for comparison. The point at which the culture reaches a doubling is indicated, as well as the linear fit during steady-state growth following recovery. **B.** Dynamic resistance is calculated as the drug concentration that causes a 2-hour delay in the time to reach one doubling following

drug exposure. Steady-state resistance is calculated as the drug concentration that reduces steady-state growth to half of the growth rate under no drug. Curves were fit with second order polynomials.

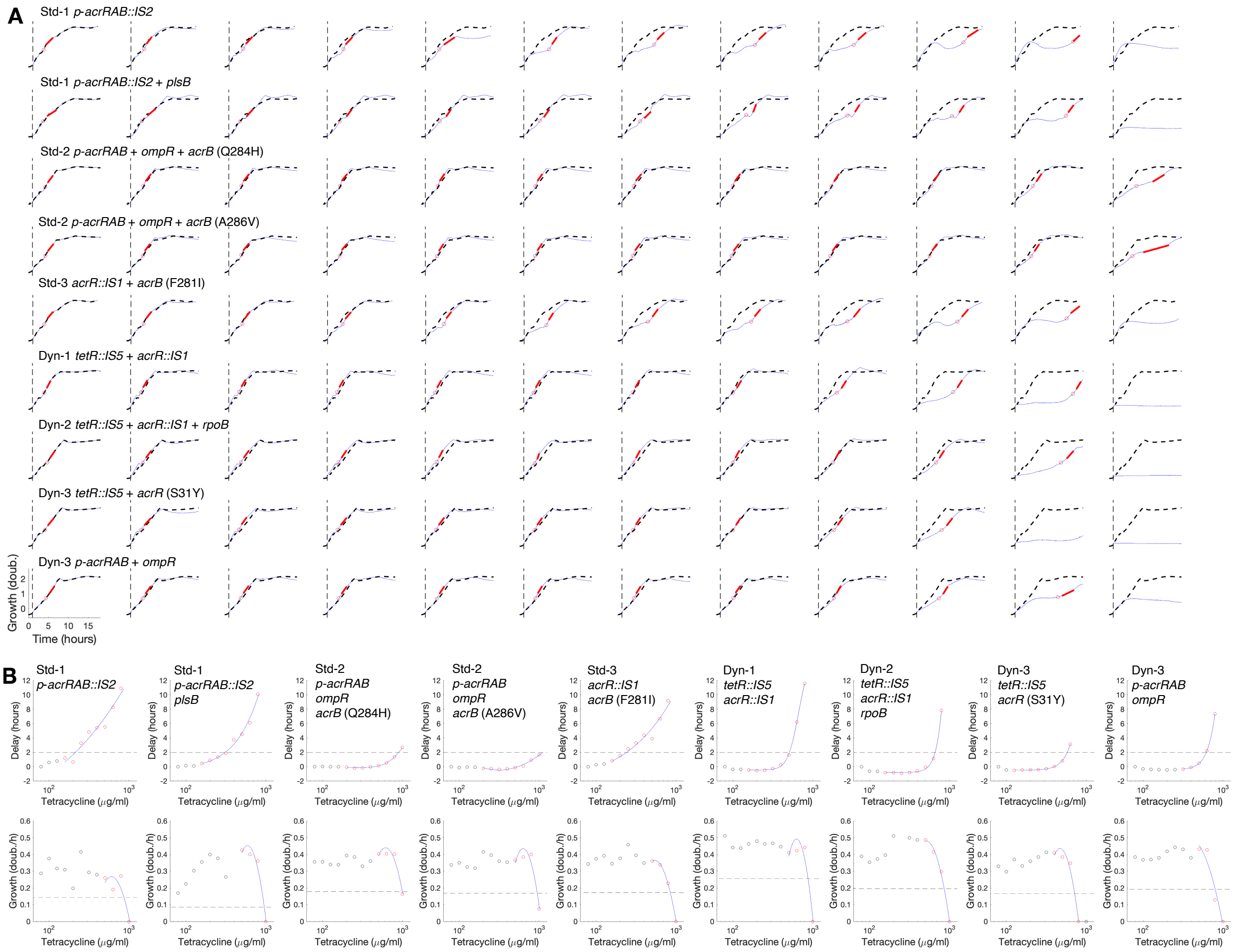

Figure S6. Calculation of steady-state and dynamic resistances for select mutants. A. Growth curves of select isolates picked from the final populations. Plots are $\log_2$(OD) over 20 hours. Vertical dashed line indicates addition of tetracycline picked from a gradient of zero in the first column then 100 to 1000 µg/ml in columns 2 to 12. The corresponding growth curve with zero drug is included in a dashed line for comparison. The point at which the culture reaches a doubling is indicated, as well as the linear fit during steady-state growth following recovery. **B.** Dynamic resistance is calculated as the drug concentration that causes a 2-hour delay in the time to reach one doubling following drug exposure. Steady-state resistance is calculated as the drug

concentration that reduces steady-state growth to half of the growth rate under no drug. Curves were fit with second order polynomials.

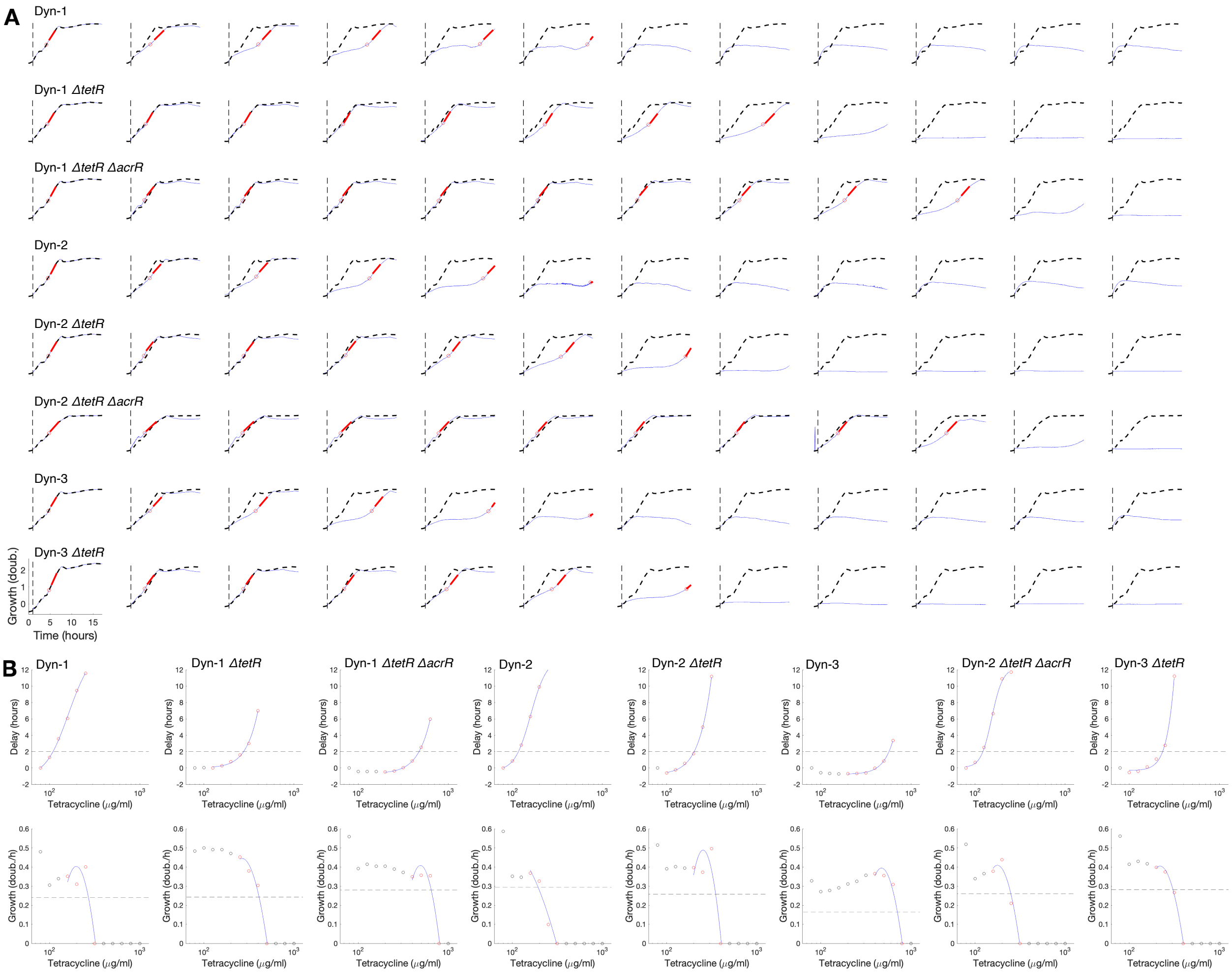

Figure S7. Calculation of gains in steady-state and dynamic resistances upon loss of TetR function. A. Growth curves of isolates picked in the same day, with and without transposon insertion in TetR, for each Dynamic population. Further acquisitions of AcrR mutations are also included. Plots are $\log_2$(OD) over 20 hours. Vertical dashed line indicates addition of tetracycline picked from a gradient of zero in the first column then 100 to 1000 μg/ml in columns 2 to 12. The corresponding growth curve with zero drug is included in a dashed line for comparison. The point at which the culture reaches a doubling is indicated, as well as the linear fit during steady-state growth following recovery. **B.** Dynamic resistance is calculated as the drug concentration that causes a

2-hour delay in the time to reach one doubling following drug exposure. Steady-state resistance is calculated as the drug concentration that reduces steady-state growth to half of the growth rate under no drug. Curves were fit with second order polynomials.
